# Forebrain overexpression of CaMKII abolishes cingulate long term depression and reduces mechanical allodynia and thermal hyperalgesia

**DOI:** 10.1186/1744-8069-2-21

**Published:** 2006-06-15

**Authors:** Feng Wei, Guo-Du Wang, Chao Zhang, Kevan M Shokat, Huimin Wang, Joe Z Tsien, Jason Liauw, Min Zhuo

**Affiliations:** 1 Department of Physiology, Faculty of Medicine, University of Toronto, 1 King's College Circle, University of Toronto, Toronto, M5S 1A8, Canada; 2 Department of Cellular and Molecular Pharmacology, University of California San Francisco, San Francisco, California 94130-0450, USA; 3 Center for Systems Neurobiology, Departments of Pharmacology and Biomedical Engineering, Boston University, Boston, MA 02118, USA; 4 Dept. of Biomedical Sciences, University of Maryland Dental School, Baltimore, MD 21201, USA

## Abstract

Activity-dependent synaptic plasticity is known to be important in learning and memory, persistent pain and drug addiction. Glutamate NMDA receptor activation stimulates several protein kinases, which then trigger biochemical cascades that lead to modifications in synaptic efficacy. Genetic and pharmacological techniques have been used to show a role for Ca^2+^/calmodulin-dependent kinase II (CaMKII) in synaptic plasticity and memory formation. However, it is not known if increasing CaMKII activity in forebrain areas affects behavioral responses to tissue injury. Using genetic and pharmacological techniques, we were able to temporally and spatially restrict the over expression of CaMKII in forebrain areas. Here we show that genetic overexpression of CaMKII in the mouse forebrain selectively inhibits tissue injury-induced behavioral sensitization, including allodynia and hyperalgesia, while behavioral responses to acute noxious stimuli remain intact. CaMKII overexpression also inhibited synaptic depression induced by a prolonged repetitive stimulation in the ACC, suggesting an important role for CaMKII in the regulation of cingulate neurons. Our results suggest that neuronal CaMKII activity in the forebrain plays a role in persistent pain.

## Introduction

Ca^2+^/calmodulin-dependent kinase II (CaMKII) is a key molecule involved in regulating glutamatergic synaptic transmission and learning and memory [1,2]. Several lines of evidence support a role for CaMKII in synaptic plasticity and memory (see [2] for review). The ability of CaMKII to autophosphorylate, which prolongs activation in a CaM independent manner, supports an important role for CaMKII in synaptic plasticity [2]. A point mutation in the CaMKII gene that blocks autophosphorylation abolished LTP and impaired spatial memory in mice [3]. While the autophosphorylation and dephosphorylation of CaMKII support the hypothesis that it can act as a "molecular switch" in plasticity and memory, recent studies favor a more sophisticated frequency-dependent biphasic modulation of CaMKII in learning-related synapses. Transgenic mice that overexpress a calcium independent form of CaMKII in forebrain areas demonstrated a shift of frequency-dependent responses to repetitive stimulation [4]. Consistently, these mice displayed an impairment in spatial memory [5]. The role of CaMKII in central synaptic plasticity is not limited to the hippocampus and spatial memory. In the cortex, αCaMKII activity plays a role in long-term or permanent memory and cortical LTP [6,7].

Glutamatergic synapses involved in sensory transmission can undergo learning-like plastic changes [8-11]. Long-term changes in plasticity along sensory transmission pathways, including the spinal cord dorsal horn and cortical neurons, have been reported [12,13]. A role for CaMKII in experience-dependent plasticity was shown in the spinal cord dorsal horn, and spinal CaMKII is involved in the development of persistent pain [14,15]. However, no study has determined if forebrain CaMKII plays a role in the development of persistent pain. The present study takes advantage of recently developed CaMKII transgenic mice that use genetic and chemical techniques to spatially and temporally restrict the expression of CaMKII [16], We tested if the overexpression of CaMKII in the forebrain affected neuronal properties and synaptic plasticity in the anterior cingulate cortex (ACC), and then carried out behavioral studies to see if responses to peripheral noxious stimuli and tissue injury were affected in forebrain CaMKII overexpressing mice. Synaptic plasticity in the ACC may serve as a cellular model for studying the roles of CaMKII in forebrain structures and behavioral alternations to persistent pain may be due to changes in these forebrain neurons.

## Methods

### Animals

CaMKII F89G transgenic male mice (8–12 weeks) were provided by Dr. Tsien's lab (see [16]). The transgene founders were produced by pronuclear injection of linearized DNA into B6/CBA F1 zygotes, and then intercrossed with B6/CBAF1. The CaMKII F89G transgene expression vector was constructed by inserting the 2.8-kb *Not*I fragment of the CaMKII F89G transgene into the unique *Not*I site of pMM279 containing a 8.5-kb CaMKII promotor sequence. Genotyping of the CaMKII F89G transgene was conducted by PCR. Both wild-type and transgenic mice were well groomed and showed no signs of abnormality or any obvious motor defects. As it was impossible to visually distinguish between transgenic and wild-type mice, experimenters were blind to the genotype. The experimental protocols were approved by the Animal Studies Committee at Washington University and University of Toronto.

### Slice electrophysiology

Transverse slices of the ACC were rapidly prepared and maintained in an interface chamber at 28°C, where they were subfused with artificial cerebrospinal fluid (ACSF) consisting of (in mM) 124 NaCl, 4.4 KCl, 2.0 CaCl_2_, 1.0 MgSO_4_, 25 NaHCO_3_, 1.0 Na_2_HPO_4_, and 10 glucose, bubbled with 95% O_2 _and 5% CO_2_. Slices were kept in the recording chamber for at least two hr prior to the start of experiments. A bipolar tungsten stimulating electrode was placed in layer V of the ACC, and extracellular field potentials were recorded using a glass microelectrode (3–12 MΩ, filled with ACSF) placed in layer II/III. Five trains of theta burst stimulation (TBS), which consisted of five bursts (four pulses at 100 Hz) of stimuli delivered every 200 ms at the same intensity, were applied to induce LTP. A low-frequency, prolonged stimulation (1 Hz, 15 min) was used to induce LTD. Synaptic responses were elicited at 0.02 Hz. In some experiments, intracellular recordings were also performed with glass microelectrodes filled with 2% Neurobiotin-2 M potassium chloride (80–200 MΩ). After electrophysiological characterization, cells were injected with neurobiotin (+3.0 nA, 150 ms, 3.3 Hz for 5 min). The slices then fixed in 4% paraformaldehyde 30 min after the injection. Morphological procedures were used to stain and identify the recorded cells.

### Acute behavioral responses to noxious stimuli

The tail-flick reflex was evoked by focused, radiant heat applied to underside of the tail. The latency to reflexive removal of the tail away from the heat was measured by a photocell timer to the nearest 0.1 sec. The hot-plate (HP) reflex was measured on a metal plate at 55°C (Columbia Instruments). Nociceptive responses included licking or lifting of a hindpaw, or jumping. All mice showed responses within 20 sec. They were removed from the chamber immediately after the first response. In some experiments, the HP response was measured at different temperatures (50 or 52°C). All behavioral tests were performed at 10 min intervals. The baseline response latency was an average of three or four measurements.

### Inflammatory pain and measurement of allodynia/hyperalgesia

Formalin (5%, 10 μl) was injected subcutaneously into the dorsal side of a hind paw. The total time spent licking or biting the injected hind paw was recorded and averaged for each 5 min interval over the course of 2 hr. For inflammatory pain, complete Freund's adjuvant (CFA, 50% in saline, 10 μl; Sigma) was injected into the dorsal surface of the left hind paw. After 24 h and 72 h, animals were placed in individual plastic boxes and allowed to adjust to the environment for 1 h. Using the up-down paradigm, mechanical sensitivity was assessed with a set of von Frey filaments (Stoelting; Wood Dale, Illinois) modified so that the filament extended in parallel to the rod. The up-down paradigm method was used to determine the mechanical threshold. For the measurement of mechanical allodynia, based on preliminary experiments that characterized the threshold stimulus, the No. 2.44 filament (0.036 gm force) innocuous for untreated wild-type mouse but representing 50% of the threshold force in animals with injection of CFA, was used to detect mechanical allodynia [16,18]. The filament was applied to the point of bending 6 times each to the dorsal surfaces of the left and right hindpaws. Positive responses included prolonged hindpaw withdrawal followed by licking or scratching. For each time point, the percent response frequency of hindpaw withdrawal was expressed as (number of positive responses)/6 × 100 per hindpaw.

Heat hyperalgesia was measured by recording the withdrawal latency of a single hind paw from a radiant heat source (IITC Life Sciences instrument, Woodland Hills, Calif). Mice were placed in Plexiglas restrainers on an elevated platform with a clear glass top. A radiant heat source with an "active intensity" of 30 (active intensity is the intensity of light source as coined by the manufacturer) was used as the stimulus. The heat source was positioned on the plantar skin of a hind paw. A built in timer starts when the light beam is switched on. When the animal withdrew its paw abruptly, the heat source and the timer were stopped simultaneously. The duration in seconds from the start of heat application to the paw withdrawal was measured as the paw withdrawal latency.

### Pharmacology and drug administration

Chemical synthesis and tritiation of 1-Naphthylmethyl (1NM-PP1) was described previously [16]. Briefly, the ATP-binding pocket of αCaMKII kinase was enlarged via silent mutation. 1NM-PP1 was designed to fit only this enlarged pocket and not the unmodified pocket of native αCaMKII. By using αCaMKII promoter-driven construct, we were able to selectively over express αCaMKII in forebrain neurons. The over expressed αCaMKII-F89G activity could be inhibited by noninvasive oral intake of 1NM-PP1 (5 μM in drinking water) for more than 24 hours (see [14]). The complete inhibition of αCaMKII-F89G activity can be maintained as long as the 1NM-PP1-containing water is provided. In some experiments, slices from wild-type littermates taking 1NM-PP1-containing drinking water were used as controls.

### Data analysis

Data are presented as the mean ± SEM. Statistical comparisons were made with the use of analyses of variance (ANOVAs: Newman-Keuls tests for post hoc comparison) or Student's t-test. P < 0.05 was considered significant.

## Results

In general, αCaMKII-F89G transgenic (Tg) mice were visually indistinguishable from wild-type mice. Our previous study used biochemical and pharmacological techniques to screen five different lines of transgenic mice, and the CaMKII Tg-1 line was found to have the highest transgenic mRNA expression. CaMKII overexpression was limited to the forebrain areas, including the ACC, hippocampus, and amygdala, using a αCaMKII promoter-driven construct (see Methods and [14]). No CaMKII overexpression was detected in the hindbrain or spinal cord [16]. Furthermore, biochemical experiments showed that calcium-dependent CaMKII activity was increased by 2.6 fold in transgenic mice compared to wild-type mice. No changes in other protein kinase such as β CaMKII or CaMKIV were found. Administration of 0.5 μM 1NM-PP1 (a compound that inhibits transgenic CaMKII, see methods) in vitro or systemic application of 1NM-PP1 in Tg-1 mice was found to inhibit overexpressed activity without effecting basal CaMKII in wild-type mice. Thus we chose to use the Tg-1 line of transgenic αCaMKII-F89G mice in the present study.

**Figure 1:**
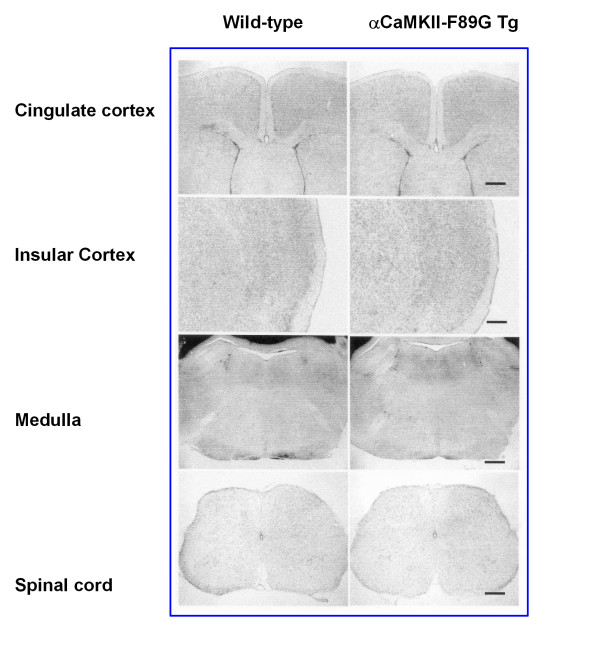
**Brain morphology of wild-type and αCaMKII-F89G transgenic mice. **Coronal sections showed no detectable morphological differences in the ACC, insular cortex, rostroventral medulla (RVM) and spinal cord dorsal horn. Scale bar: 250 μm (ACC, insular cortex and RVM), and 100 μm (spinal dorsal horn).

**Figure 2:**
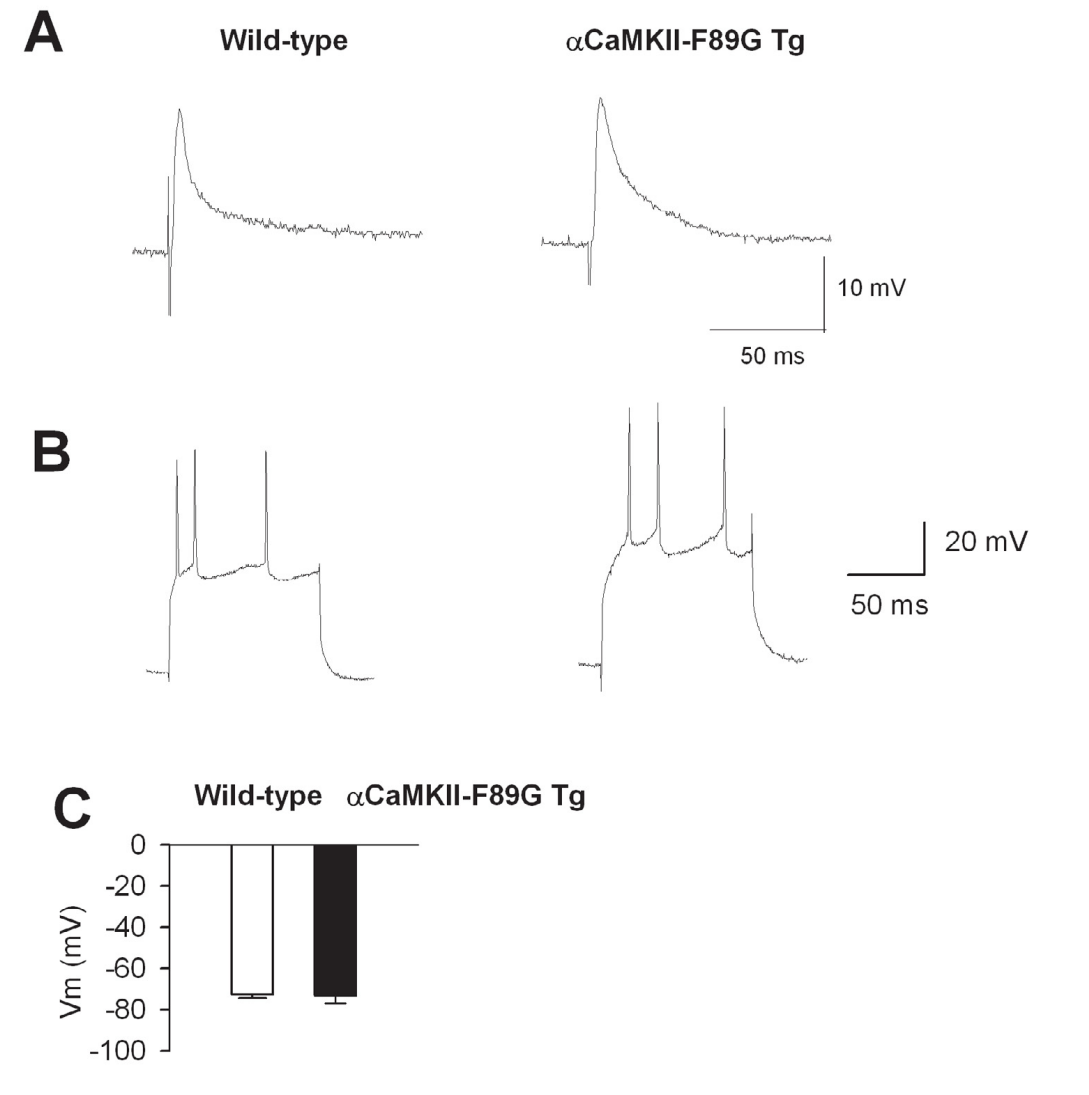
**Comparison of neuronal properties in wild-type and αCaMKII-F89G transgenic mice. **A. Evoked synaptic responses recorded to focal stimulation in the ACC of wild-type and αCaMKII-F89G transgenic mice. B. Action potentials of ACC neurons from wild-type and αCaMKII-F89G transgenic mice. C. Resting membrane potentials from ACC neurons of wild-type and αCaMKII-F89G transgenic mice. Note: no significant difference was found between wild-type and αCaMKII-F89G transgenic mice.

We first performed anatomical experiments in wild-type and αCaMKII-F89G transgenic mice to check whether the gross development of several sensory related brain areas were affected. Analysis of serial coronal sections, examined by light microscopy, showed no visual detectable morphological differences in the ACC, insular cortex, brainstem, and spinal dorsal horn between CaMKII Tg and WT mice (Fig. 1). Next we asked whether the neuronal spiking properties of ACC neurons might be affected in the transgenic mice. We performed intracellular recordings from ACC neurons (Fig. 2A–C). Similar to results found in the hippocampus, we did not find any significant changes in baseline synaptic responses in ACC neurons (wild-type, n = 16 neurons/10 mice; transgenic, n = 10 neurons/6 mice). No significant changes were observed in neuronal spiking in response to direct current injection (wild-type, n = 12 neurons/8 mice; transgenic, n = 9 neurons/6 mice). Resting membrane potentials were identical between cells recorded from wild-type (n = 14 neurons/10 mice) and transgenic mice (n = 12 neurons/6 mice). Furthermore, the current threshold for eliciting the first spike was similar in ACC neurons from wild-type and transgenic mice (n = 3–6 neurons for each group) and the number of spiking induced by injection of 0.3 nA was also identical (n = 6–10 cells). These results indicate that αCaMKII overexpression did not cause changes in the intrinsic neuronal electrophysiological properties of ACC neurons.

**Figure 3:**
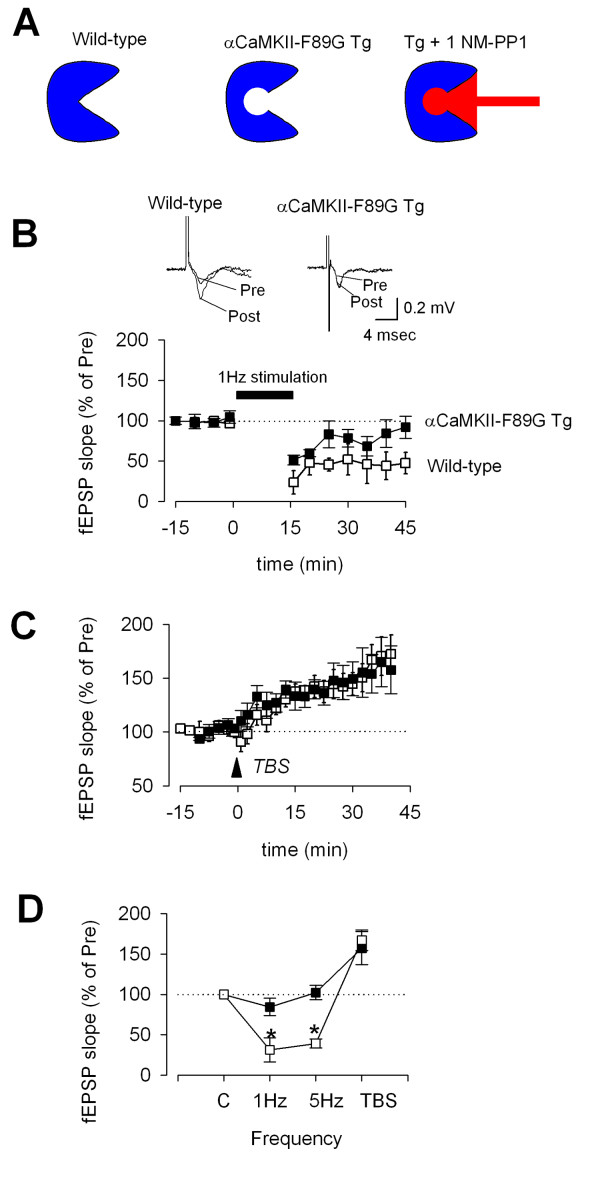
**Long-term potentiation (LTP) and long-term depression (LTD) in ACC slices of wild-type and αCaMKII-F89G transgenic mice. **A. Model for the design of αCaMKII-F89G transgenic mice and the selective chemical inhibitor. B. Prolonged low frequency stimulation (1 Hz for 15 min) induced LTD of synaptic responses in the ACC of wild-type mice. However, LTD was abolished in slices of αCaMKII-F89G transgenic mice. Inset: traces of field EPSPs recorded before (Pre) and after [22] LTD induction in wild-type and αCaMKII-F89G transgenic mice. C. Similar LTP was induced by theta burst stimulation (TBS) in ACC slices of wild-type and αCaMKII-F89G transgenic mice. D. Summarized results of frequency-dependent responses in wild-type and αCaMKII-F89G transgenic mice. The average field EPSPs (% of control) of last 5 min recording (40–45 min in A) were used for the plot. * P < 0.05 significantly different from wild-type mice.

CaMKII activity is important for synaptic plasticity. In a previous study of αCaMKII-F89G transgenic mice, we found that αCaMKII overexpression selectively affected the frequency-response relationship of synaptic plasticity in the CA1 region of the hippocampus [16]. In addition to enhanced LTP induced by strong tetanic stimulation, synaptic responses in response to repetitive stimulation at lower frequencies were also altered [16]. To study if cingulate plasticity may also be affected in these mice, we decided to investigate both LTP and LTD in ACC slices of wild-type and transgenic mice. We did not detect changes in synaptic potentiation induced by theta burst stimulation in ACC slices of transgenic mice (mean 157.2 ± 20.4% of control; n = 6 slices/5 mice) as compared to wild-type mice (mean 167.2 ± 12.8% of control, n = 12 slices/10 mice; P = 0.346) (Fig. 3C). To detect possible changes in frequency-dependent responses, we examined the effect of prolonged repetitive stimulation in cingulate slices. We previously showed that low-frequency repetitive stimulation at 1 Hz for 15 min induced LTD in the ACC of adult rats [9]. Furthermore, repetitive stimulation at 3 or 5 Hz stimulation also induced LTD of synaptic responses in ACC slices [9]. As shown in Fig. 3B, we found that 1 Hz stimulation (15 min) induced robust LTD in the ACC of wild-type mice (8–12 weeks old) (mean 35.7 ± 14.1%, n = 6 slices/6 mice, P < 0.05 compared with baseline). Similar to ACC in adult rats, 5 Hz stimulation for 3 min also produced long-lasting depression of synaptic responses in ACC slices (mean 39.1 ± 5.5%, n = 5 slices/5 mice; P < 0.001 as compared with baseline) (Fig. 3D). We then tested if forebrain overexpression of CaMKII affects synaptic LTD in the ACC. As shown in Figure 3B, we found that LTD induced by 1 Hz stimulation (15 min) was significantly reduced or completely abolished in transgenic mice (mean 84.4 ± 10.7%, n = 6 slices/6 mice; P < 0.01 as compared with LTD in wild-type mice). Similarly, in transgenic mice, 5 Hz stimulation did not induce any synaptic depression (mean 102.3 ± 8.9%, n = 5 slices/5 mice, Fig. 3; P < 0.001 as compared with wild-type mice) (Fig. 3D).

**Figure 4:**
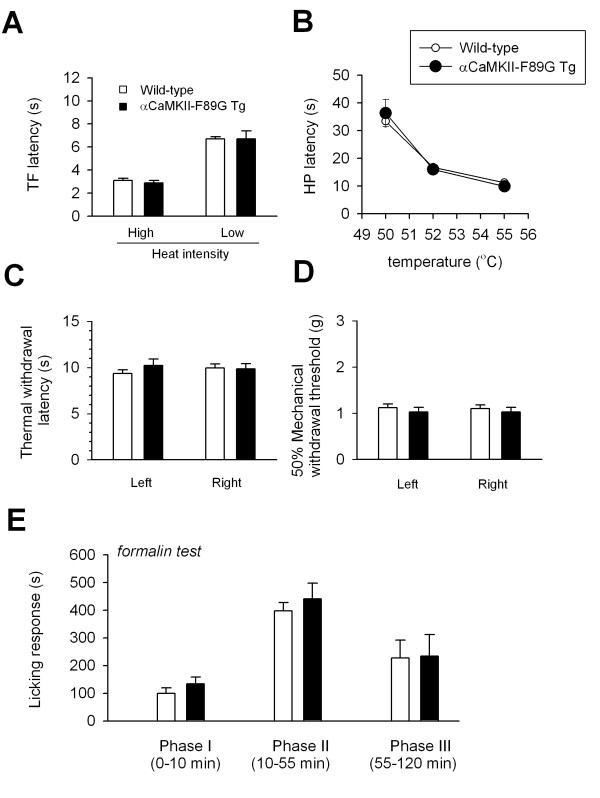
**Behavioral nociceptive responses to noxious heat, mechanical pressure and formalin injection were not altered in αCaMKII-F89G transgenic mice. **A-B. Behavioral nociceptive responses to noxious heating in the tail-flick test at two different heating intensities (A) and the hot-plate test at three different temperatures (50, 52, 55°C) (B). No significant differences were found between wild-type and αCaMKII-F89G transgenic mice in any test. C-D. No significant differences were found in hind paw withdrawal responses to noxious heating or mechanical pressure. E. Behavioral nociceptive responses to hindpaw formalin injection, plotted in 5-min intervals, in wild type mice as compared to αCaMKII-F89G transgenic mice. No significant difference was found between wild-type and transgenic mice.

Results described above indicate that overexpressing CaMKII in the forebrain causes selective changes in synaptic LTD without causing any obvious anatomical abnormality or change in neuronal excitability. These findings suggest that αCaMKII-F89G transgenic mice may serve as an excellent model for investigating the role of CaMKII in forebrain plasticity. Since forebrain structures play a key role in sensory perception and plasticity, we next wanted to determine if forebrain CaMKII plays a role in behavioral responses to sensory stimuli and injury. To study the behavioral responses to acute noxious stimuli, we performed the tail-flick and hot-plate tests. We found that behavioral responses to noxious thermal stimuli were similar between wild-type and transgenic mice (Fig. 4, tail-flick test: wild-type, n = 6 mice; transgenic mice, n = 7 mice; thermal withdrawal: wild-type, n = 10 mice, transgenic mice, n = 6 mice), indicating that enhanced CaMKII activity in the forebrain did not significantly affect acute behavioral responses to noxious stimuli. To detect possible temperature-dependent changes in hot-plate responses, we measured responses at additional temperatures (50 and 52°C). Again, we did not find any significant difference (n = 12 mice for wild-type mice, n = 11 for transgenic mice) (Fig. 4B). We also measured hindpaw withdrawal to mechanical pressure and found that mechanical withdrawal thresholds were not affected in transgenic mice (n = 4 mice for each group, Fig. 4D). These results consistently demonstrate that behavioral responses to acute noxious thermal and mechanical stimuli were not affected by enhanced forebrain CaMKII activity.

**Figure 5:**
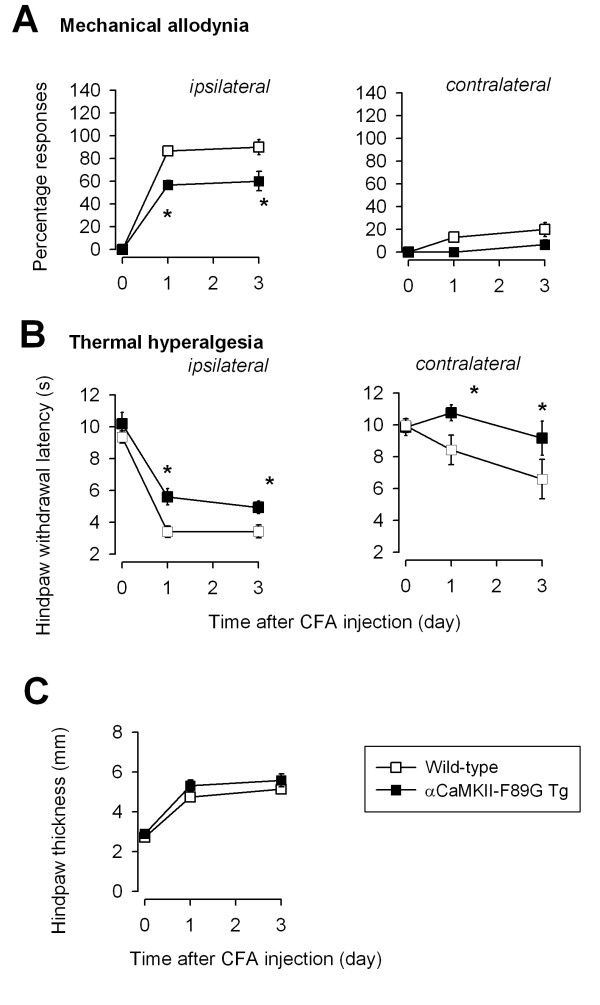
**Reduction of behavioral sensitization (allodynia) and hyperalgesia after CFA injection in αCaMKII-F89G transgenic mice. **A. The behavioral responses of animals to a non-noxious mechanical stimulus (No. 2.44 von Frey fiber, which elicited no response before a dorsal hindpaw CFA injection), were recorded 1 and 3 days after injection. The data were plotted as percentage positive responses to stimulation of the ipsilateral or contralateral hind paw of wild-type and αCaMKII-F89G transgenic mice. Inflammation induced less behavioral allodynia in αCaMKII-F89G transgenic mice then wild-type mice (P < 0.05; indicated by *). B. The behavioral responses of animals to a noxious thermal stimulus were recorded 1 and 3 days after the injection. The data were plotted as response latency to stimulation of the ipsilateral or contralateral hind paw of wild-type and αCaMKII-F89G transgenic mice. Inflammation induced less hyperalgesia in αCaMKII-F89G transgenic mice then wild-type mice (P < 0.05; indicated by *). C. Hindpaw edema was measured with a fine caliper in wild-type and αCaMKII-F89G transgenic mice.

NMDA receptors and their related signaling pathways in the forebrain (including the ACC) have been implicated in injury-related behavioral sensitization [17-19]. Thus, it is possible that CaMKII contributes to behavioral responses to tissue injury and inflammation, a long-lasting form of behavioral sensitization. The formalin test measures spontaneous responses to tissue injury and inflammation [17,20]. Formalin-induced behavioral responses consist of three phases and depend on NMDA receptors at different levels of the brain [10,21]. We tested formalin-induced nociceptive responses in wild-type and CaMKII transgenic mice and found that all the three phases did not differ between transgenic (n = 7 mice) and wild-type (n = 13 mice) mice (Fig. 4E). In animals with persistent pain, behavioral sensitization to non-noxious stimuli or mechanical allodynia happens after tissue injury. We next tested the role of enhanced CaMKII activity in the development of allodynia induced by a hind paw injection of CFA (50%, 10 μl). Application of a non-noxious von Frey fiber to the dorsum of a hind paw elicited no response in untreated wild-type mice, but at one and three days after CFA injection into the dorsum of a single hind paw, mice withdrew their hindpaw in response to stimulation of the ipsilateral or, to a lesser extent, the contralateral hind paw (Fig. 5A). This mechanical allodynia was significantly reduced in transgenic mice compared to wild-type mice (n = 5 mice for each group; Fig. 5A; P < 0.01 as compared between wild-type and transgenic mice). Similar results were observed in the contralateral hind paw. In wild-type mice, we found a significant decrease in hindpaw withdrawal latencies from a noxious heat source at 1 to 3 days after CFA injection in the ipsilateral hindpaw (so called thermal hyperalgesia) (n = 5 mice), and to a lesser extent in the contralateral hind paw (Fig. 5B). However, thermal hyperalgesia was significantly reduced in CaMKII transgenic mice (n = 5 mice) compared to wild-type mice (Fig. 5B). To be sure that the differences in pain behaviors were not attributable to differences in peripheral inflammation, we measured hind paw edema in both wild-type and transgenic mice. A similar degree of inflammation was found in wild-type and transgenic mice (n = 5 mice for each group, Fig. 5C), indicating that the peripheral responses to inflammation are likely identical in these mice.

**Figure 6:**
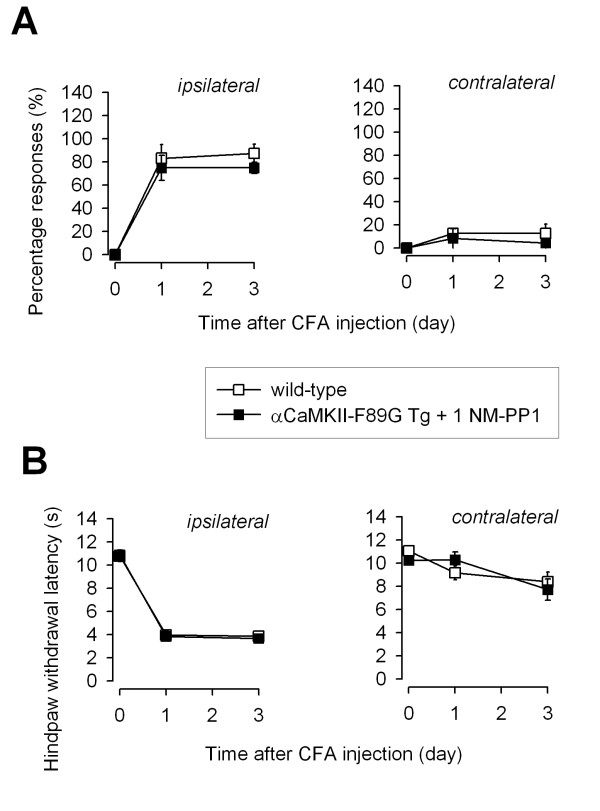
**Reduction in allodynia and hyperalgesia can be reversed by inhibiting the overexpression of CaMKII activity in αCaMKII-F89G transgenic mice. **A. One-week pretreatment with the inhibitor 1NM-PP1 reversed the reduction of behavioral allodynia in αCaMKII-F89G transgenic mice. No significant difference was found between wild-type mice and αCaMKII-F89G transgenic mice receiving 1NM-PP1 pretreatment. B. One-week pretreatment with 1NM-PP1 reversed the reduction of behavioral hyperalgesia in αCaMKII-F89G transgenic mice. No significant difference was found between wild-type mice and αCaMKII-F89G transgenic mice receiving 1NM-PP1 pretreatment.

Our results indicate that the forebrain overexpression of CaMKII significantly affected synaptic depression in the ACC in vitro and behavioral sensitization to inflammation in vivo. However, we cannot rule out the possibility that these changes in behavioral responses may due to developmental, long-term changes in CaMKII-dependent or related signaling pathways. In the present study, the chemically engineered mice provide us a chance to turn off the overexpressed CaMKII activity and examine if the observed changes can be reversed or 'rescued'. To test this, we treated CaMKII transgenic mice with the inhibitor 1NM-PP1 for several days to turn off the overexpressed CaMKII activity. We found that 1NM-PP1 treatment completely blocked the effects of CaMKII overexpression on behavioral allodynia and hyperalgesia in transgenic mice (n = 4–6 mice, see Fig. 6A and 6B, respectively). 1NM-PP1 treatment alone did not significantly affected hindpaw mechanical withdrawal thresholds (n = 6 mice).

**Figure 7:**
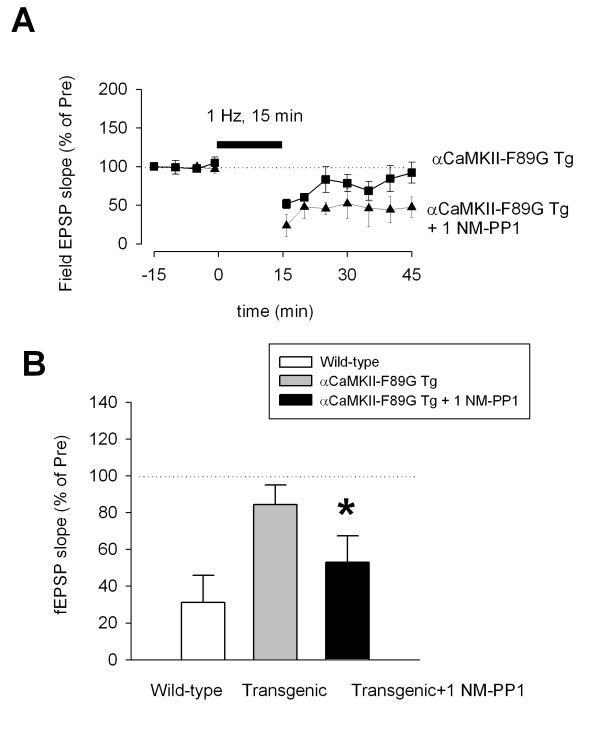
**'Rescued' LTD in αCaMKII-F89G transgenic mice by inhibiting the overexpression of CaMKII activity in αCaMKII-F89G transgenic mice. **A. One-week pretreatment with 1NM-PP1 reversed the reduction of cingulate LTD in αCaMKII-F89G transgenic mice. For comparison, the same plot of αCaMKII-F89G transgenic mice shown in Figure 3 was used. B. Summarized data for LTD in wild-type, αCaMKII-F89G transgenic mice, and αCaMKII-F89G transgenic mice with 1NM-PP1 pretreatment. The average field EPSPs (% of control) of last 5 min recording (40–45 min in A) were used for the plot. * P < 0.05 as compared with αCaMKII-F89G transgenic mice.

Finally, we tested the effects of 1NM-PP1 on synaptic plasticity to determine if reversing CaMKII over expression could 'rescue' the loss of synaptic depression induced by 1 Hz repetitive stimulation. As shown in Figure 7, we found that 1NM-PP1 rescued synaptic depression in the transgenic mice (mean 53.2 ± 11.0% of control, n = 7 slices/6 mice, P < 0.05 as compared with transgenic mice). By contrast, the inhibitor did not significantly affect baseline synaptic responses in the ACC slices (n = 6 slices/6 mice) or synaptic depression in slices of wild-type mice (mean 40.9 ± 12.1% of control, n = 5 slices/5 mice).

## Discussion

Our results provide the first evidence that CaMKII activity in the forebrain plays an important role in cingulate synaptic depression and behavioral sensitization related to persistent pain after hindpaw inflammation. While our results do not show a direct correlation between the loss of synaptic LTD in the ACC and a reduction in behavioral sensitization in CaMKII transgenic mice, we suggest that the enhanced forebrain CaMKII activity reduced behavioral sensitization to peripheral injury and that the synaptic function of CaMKII in cingulate neurons may be one mechanism contributing to this behavioral phenotype. Although we focused on the area of the ACC in the current study, we cannot rule out the possible contribution of other forebrain areas such as insular cortex and prefrontal cortex to behavioral changes in the transgenic mice. Our present findings, together with a previous report of increased pain behaviors in forebrain NR2B overexpressing mice (Wei et al, 2001), consistently suggest that behavioral sensitization after tissue injury can be regulated or modulated by neuronal activity within the forebrain areas, including the ACC. Loss of LTD in CaMKII transgenic mice may be responsible for the reduction of nociceptive responses after tissue injury. The rescuing effect of 1NM-PP1 in CaMKII transgenic mice provides strong evidence that the electrophysiological and behavioral changes observed in CaMKII transgenic mice are not due to any developmental changes induced by CaMKII overexpression.

The present results provide strong evidence that enhancing the activity of NMDA receptors or CaMKII does not have the same consequence on persistent pain despite being equally implicated in learning-related synaptic plasticity and behavioral memory. We found that forebrain overexpression of CaMKII did not affect basal nociception and behavioral nociceptive responses to subcutaneous injection of formalin. In our previous study in mice over expressing NMDA NR2B receptors in forebrain regions, we found that late behavioral nociceptive responses to formalin was selectively enhanced in transgenic mice [10]. One possible explanation for such a difference is that the over expressed NMDA NR2B receptors are inactive in normal conditions due to the magnesium blockade of NMDA receptors. However, the enhanced activity of CaMKII would tonically alter the signaling pathways within neurons, including a disruption of normal function and expression of NMDA receptors in the forebrain. Therefore, over expressed CaMKII activity within neurons may have opposite effects on certain outcomes (either at synaptic or behavioral levels) as compared with NMDA receptors.

Why was behavioral sensitization to injury reduced while synaptic depression abolished in the ACC CaMKII transgenic mice? One major hypothesis for the roles of the ACC in pain is that neuronal activity in ACC neurons code the magnitude of pain or pain-related unpleasantness [19]. LTP within the ACC caused by injury or amputation may in part encode abnormal or enhanced pain sensation [19]. The function of synaptic LTD is always thought to be the reversed form of LTP; in the case of memory, LTD is proposed to help to erase old memory and thus allow new memory to form. If we treated plastic changes in the ACC as a memory event caused by injury (e.g., CFA injection in this report), we would predict that loss of LTD in the ACC in CaMKII transgenic mice might abolish the animal's ability to 'erase' such 'bad' or 'painful' memory. Therefore, we would predict that behavioral sensitization would be more likely to be enhanced in CaMKII transgenic mice. By contrast, we found that behavioral sensitization was reduced in CaMKII transgenic mice. It is clear that more experiments are needed in the future to investigate if changes in NMDA receptor expression and function in the ACC and other areas in CaMKII transgenic mice may underlie these results. The roles of cingulate synaptic depression during the development of behavioral sensitization remain to explore.

Considering the important roles of CaMKII in neurodevelopment, it is important to confirm that the phenotypic changes found in brain slices and in nociceptive behaviors can be reversed by simply inhibiting the over expression of CaMKII. In the present study, we found that pretreatment with the inhibitor 1NM-PP1 rescued the loss of behavioral sensitization as well as synaptic depression in ACC slices. Therefore, our studies provide strong evidence for the physiological roles of CaMKII in cingulate neurons, both in terms of selectivity and regional effects.

The present new transgenic mice provide a powerful, and new tool for investigating the roles of protein kinases in the expression or maintenance of behavioral sensitization. Understanding these molecular and synaptic mechanisms hold promise for treating chronic pain in patients.
